# Phase−amplitude coupling between theta and gamma oscillations adapts to speech rate

**DOI:** 10.1111/nyas.14099

**Published:** 2019-04-24

**Authors:** Mikel Lizarazu, Marie Lallier, Nicola Molinaro

**Affiliations:** ^1^ BCBL Basque Center on Cognition, Brain and Language Donostia/San Sebastian Spain; ^2^ Laboratoire de Sciences Cognitives et Psycholinguistique, Dept d'Etudes Cognitives, ENS PSL University EHESS, CNRS Paris France; ^3^ Ikerbasque Basque Foundation for Science Bilbao Spain

**Keywords:** neural oscillations, speech rate, auditory regions, magnetoencephalography

## Abstract

Low‐ and high‐frequency cortical oscillations play an important role in speech processing. Low‐frequency neural oscillations in the delta (<4 Hz) and theta (4–8 Hz) bands entrain to the prosodic and syllabic rates of speech, respectively. Theta band neural oscillations modulate high‐frequency neural oscillations in the gamma band (28−40 Hz), which have been hypothesized to be crucial for processing phonemes in natural speech. Since speech rate is known to vary considerably, both between and within talkers, it has yet to be determined whether this nested gamma response reflects an externally induced rhythm sensitive to the rate of the fine‐grained structure of the input or a speech rate−independent endogenous response. Here, we recorded magnetoencephalography responses from participants listening to a speech delivered at different rates: decelerated, normal, and accelerated. We found that the phase of theta band oscillations in left and right auditory regions adjusts to speech rate variations. Importantly, we showed that the peak of the gamma response—coupled to the phase of theta—follows the speech rate. This indicates that gamma activity in auditory regions synchronizes with the fine‐grain properties of speech, possibly reflecting detailed acoustic analysis of the input.

## Graphical abstract

Here, we found that the phase of theta band oscillations in left and right auditory regions adjusts to speech rate variations. Importantly, we showed that the peak of the gamma response—coupled to the phase of theta—follows the speech rate. This indicates that gamma activity in auditory regions synchronizes with the fine‐grain properties of speech, possibly reflecting detailed acoustic analysis of the input.

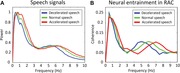

## Introduction

Recent experimental and theoretical advances in neuroscience support the idea that cortical temporal sampling plays a key role in speech processing.^1^, ^2^, ^3^ Specifically, it is proposed that oscillatory activity in the auditory cortex (AC) aligns with the temporal structure of an external rhythmic auditory input thus optimizing sensory processing. When the auditory input is a speech signal, this alignment supports the extraction of discrete linguistic units from a continuous stream of speech information. Temporal sampling is achieved in part via the entrainment of low‐frequency neural oscillations (<10 Hz) to the slow temporal modulations of speech.^4^, ^5^, ^6^ More specifically, the phase of delta (<4 Hz) and theta (4−8 Hz) oscillations in auditory regions, respectively, tracks prosodic and syllabic rhythms during speech processing.^7^, ^8^, ^9^ Interestingly, while the theta band component reflects acoustic tracking of the speech input, the delta band appears to be sensitive to language‐related processes related to syntactic properties of the speech input.^10^ Furthermore, theta band oscillations impose periods of excitation and inhibition on high‐frequency oscillations (gamma band: 25−40 Hz), a mechanism proposed to contribute to rapid decoding of high‐frequency information needed to process phonemes in the speech signal.^2^, ^11^, ^12^ Cross‐frequency phase−amplitude coupling (PAC) functions as an integrating mechanism in which the phase dynamics of low‐frequency oscillations temporally organize the amplitude of high‐frequency oscillations.^13^ Cross‐frequency PAC provides a plausible mechanism through which fine‐grain phonemic information could be grouped into syllabic units that build up words and phrases.^3^, ^14^ Supporting evidence for this model in humans comes from studies on natural speech processing: Gross and colleagues^3^ report the existence of “a nested hierarchy of auditory oscillations at multiple frequencies that match the frequency of relevant linguistic components in continuous speech.” These authors observed stronger theta−gamma coupling when participants listened to forward compared with backward speech, thereby directly linking coupling to speech processing. If cross‐frequency PAC is indeed functionally relevant for speech perception, it should not be constant over time and frequency, but dynamically adapt to the spectrotemporal features of the input. For this reason, in the present study, we focus on the frequency‐domain sensitivity of cross‐frequency PAC to speech rhythms.

Speech rate varies considerably within as well as between speakers,^15^, ^16^ and these rate changes should have a dramatic impact on the tempo of occurrences of acoustic cues within the speech envelope.^17^ Evidence for the influence of speech rate on speech comprehension accuracy has been mixed. On the one hand, some previous studies have demonstrated that the speech signal can be compressed to half of its original duration before comprehension is significantly affected.^18^, ^19^ In the same vein, Nourski *et al*.^20^ reported that speech rate is not a limiting factor in accurate speech comprehension. Overall, the aforementioned studies suggest that speech comprehension is not necessarily speech rate−dependent. On the other hand, given that listeners have limited auditory (echoic) memory for previously heard speech, it is likely that processing higher level content must proceed more quickly when speech is time‐compressed and slow down when it is expanded.^21^^−^ 23 Yet, whether and how neural processes required for speech comprehension are modulated when listeners attend faster or slower speech rates remains unclear. Low‐frequency auditory cortical entrainment has been shown to track the tempo of the speech input in a number of studies.^24^, ^25^, ^26^, ^27^ For instance, Pefkou *et al*.^27^ observed that the phase patterns of theta band oscillations consistently follow the syllabic rate for compressed speech. They also found that gamma power was sensitive to the tempo of perceived speech, showing power decreases at higher compression rates. Importantly, optimal cortical entrainment to different speech rates affects word recognition: Kösem *et al*.^26^ found that listening to sentences with altered speech rates modulated low‐frequency auditory entrainment in the theta band and this, in turn, influenced how a final word—that was lexically ambiguous due to a sound with an intermediate duration between a minimal phonetic pair—was semantically interpreted. Dilley and Pitt^28^ provided behavioral evidence that lexical identification depends on the speech rate of the overall context of an utterance. This means that comprehension of the speech signal is somehow affected by speech rate and more specifically by neural entrainment to that specific rate.

Based on this recent evidence, we reasoned that if cortical entrainment constrains speech perception, then not only low‐frequency entrainment but, more generally, the whole hierarchy of nested oscillatory patterns should flexibly adapt to the rate of the input.^14^ Word recognition is based on decoding fine‐grain temporal speech information that conveys acoustic cues necessary for the identification of individual phonemes. However, it is not clear whether high‐frequency oscillatory gamma activity (modulated by low‐frequency oscillators) adapts to the speech rate of the input or not. On the one hand, it is possible that the frequency peak of nested gamma band activity does not change its frequency peak depending on the speech rate. In fact, gamma‐band activity in auditory regions during speech processing has been associated with postperceptual processing of higher level linguistic properties.^29^, ^30^ This would imply that gamma oscillations should not necessarily support a processing mechanism that directly depends on the acoustic spectrotemporal properties of the external input. On the other hand, if gamma peak changes do depend on the speech rate of the external input, this would support the hypothesis of a speech rate−dependent gamma response directly involved in phonemic sampling. Evidence for dynamic adaptation of this gamma neural mechanism via cross‐frequency PAC is still lacking.^14^ Note that the finding that EEG gamma power reduces with increasing compression rates is not conclusive since this could also be explained by the limited spectral sensitivity of the EEG signal at such high frequencies.^27^ However, if speech rate−dependent gamma sampling could be confirmed, this would provide the strongest evidence to date for the role of hierarchical neural mechanisms in the decomposition of acoustic cues in the speech signal. More generally, it would support models proposing that high‐frequency oscillations are involved in sampling the acoustics of speech while being modulated by low‐frequency oscillatory components.^2^, ^3^, ^14^

To address this issue, we recorded magnetoencephalography (MEG) signals from participants listening to a speech at various rates: decelerated, normal, and accelerated. First, we analyzed how the neural entrainment mechanism adjusted to speech rate variations. With this aim in mind, we measured coherence between low‐frequency oscillations and speech signals for each condition. Second, we analyzed whether the coupling between low‐ and high‐frequency neural oscillations was tuned to speech rate variations. We used mutual information (MI) analysis to estimate the linear and nonlinear dependencies between low‐ and high‐frequency oscillations during the processing of each speech condition. If nested gamma oscillations reflect a fine‐grain sampling of the speech input, we expect the frequency peak of this high‐frequency component to be modulated by speech rate. However, the absence of such gamma response modulation (i.e., the gamma peak is not affected by speech rate) would call for a revision of current models of the hierarchical oscillatory sampling of speech.

## Methods

### Subjects

Seventeen (nine females) participants took part in the present study (age range: 17.1−44.1 years; *M* = 30.8; SD = 9.7). All participants were Spanish monolinguals, reported no hearing impairments, and were right‐handed. The present experiment was undertaken with the understanding and written consent of each participant (or the legal tutor of each child below 18 years of age). The ethical committee of the Basque Center on Cognition, Brain and Language (BCBL) approved the experiment (following the principles of the Declaration of Helsinki) and each participant signed an informed consent form.

### Stimuli and procedure

Three types of speech stimuli were used: (1) normal speech, (2) accelerated speech, and (3) decelerated speech. Forty meaningful sentences produced by a Spanish native female comprised the normal speech sample. The speakers were instructed to read each sentence as clearly and naturally as possible. The original speech was digitized at 44.1 kHz using a digital recorder (Marantz® PMD670) and audio files (*.wav) were segmented using Praat. The duration of the normal sentences ranged from 7.42 to 12.65 s (*M* = 9.9; SD = 1.13). The total duration of the normal speech was 6.6 min and the number of words per minute (wpm) was 173.94. This rate is within the optimal wpm range (170−190 wpm) for speech comprehension in Spanish.^31^ This normal speech was then accelerated or decelerated by changing the tempo of the audio signal using Audacity (SoundTouch Audio Processing Library by Olli Parviainen, https://www.surina.net/soundtouch/), and ensuring that the pitch of the stimulus remained unaltered. We applied a speedup factor of 1.25 and a slowdown factor of 0.75 to obtain accelerated and decelerated sentences, respectively. The total duration of the accelerated speech was 5.51 min and the number of wpm was 208.35. The total duration of the decelerated speech was 8.25 min and the number of wpm minute was 139.15.

During MEG recording, sentences were presented auditorily to the participants at 75−80‐decibel (dB) sound pressure level. Each trial began with a 1‐s auditory tone (at 500 Hz tone) followed by a 2‐s silence, and then the sentence was presented. A comprehension question about the content of the most recent sentence stimulus was presented auditorily 2 s after the end of each sentence. These yes/no questions mainly referred to the overall semantic meaning of the last sentence and avoided the repetition of lexical items used in the sentence so as to ensure that the response reflected participants’ overall semantic interpretation of the message. While listening to the sentence, participants were asked to fixate a white‐color sticker on the switched‐off screen. Participants answered the question by pressing the corresponding button (yes/no). After their response, the next trial was presented. Response hands (right/left) for yes/no responses were counterbalanced across participants and the presentation order of the sentences was randomized. Participants were asked to avoid head movements and to try to blink only during time periods between sentences. Stimuli were delivered using Presentation® software (http://www.neurobs.com/).

After the presentation of the stimuli, subjects were asked to sit as still as possible with their eyes closed. Approximately 5 min of resting state, MEG activity was recorded from each participant.

### Data acquisition

MEG data were acquired in a magnetically shielded room using the whole‐scalp MEG system (Elekta Neuromag®, Helsinki, Finland) installed at the BCBL (http://www.bcbl.eu/bcbl-facilitiesresources/meg/). The system is equipped with 102 sensor triplets (each comprising a magnetometer and two orthogonal planar gradiometers) uniformly distributed around the head of the participant. Head position inside the helmet was continuously monitored using four head position indicator coils. The location of each coil relative to the anatomical fiducials (nasion, and left and right preauricular points) was defined with a 3D digitizer (Polhemus Fastrak, Colchester, VA). This procedure is critical for head movement compensation during the data recording session. Digitalization of the fiducials plus ∼100 additional points evenly distributed over the scalp of the participant were used during subsequent data analysis to spatially align the MEG sensor coordinates with each participant's T1 magnetic resonance brain images acquired on a 3T MRI scan (Siemens Medical System, Erlangen, Germany). MEG recordings were acquired continuously with a bandpass filter at 0.01−330 Hz and a sampling rate of 1 kilohertz. Eye movements were monitored with two pairs of electrodes in a bipolar montage placed on the outer cantus of each eye (horizontal electrooculography (EOG)) and above and below the right eye (vertical EOG).

### Data preprocessing

To remove external magnetic noise from the MEG recordings, data were preprocessed offline using the signal‐space‐separation method implemented in Maxfilter 2.1 (Elekta Neuromag).^32^ MEG data were also corrected for head movements, and substitutions were made for bad channels using interpolation algorithms implemented in the software. Subsequent analyses were performed using Matlab R2010 (Mathworks®, Natick, MA). Heartbeat and EOG artifacts were detected using independent component analysis (ICA) and linearly subtracted from recordings. The ICA decomposition was performed using the Infomax algorithm implemented in the Fieldtrip toolbox.^33^

### Source activity estimation

Using the minimum‐norm estimate (MNE) suite, the digitized points from the Polhemus were coregistered to the skin surface. Individual T1‐weighted MRI images were segmented into scalp, skull, and brain components using the segmentation algorithms implemented in FreeSurfer (Martinos Center of Biomedical Imaging, MQ). Leadfield computation was based on a three‐shell volume conductor model using a 5‐mm grid defined on the template (MNI) brain. The template grid was transformed into individual headspace using the linear space transformation algorithm implemented in Statistical Parametric Mapping (SPM8, Wellcome Department of Cognitive Neurology, London, UK). The noise covariance matrix was estimated from the empty room data acquired right before bringing the subject into the MEG room. We used the noise covariance matrix to whiten the forward matrix and the data.^34^, ^35^ The cortical sources of the MEG signals were estimated using L2 MNE.^36^

For further analysis, brain signals from predefined regions of interest (ROIs) were selected. The ROIs included the left and right auditory cortex (LAC and RAC; Brodmann areas (BA) 41 and BA 42). These regions were selected from the 3D Brodmann Atlas provided with MRIcron software (available at http://www.mccauslandcenter.sc.edu/mricro/mricron). Figure S1 (online only) shows the location of the ROIs in MNI space. The focus of the present paper was on the role of gamma band activity in speech processing and for this reason, we confined our analysis to these regions.^3^

### Neural entrainment analysis

We used coherence to estimate auditory entrainment to the speech stimuli.^37^ Coherence measures the degree of phase synchronization between two signals (the speech envelope and neural oscillations in the AC) in the frequency domain. The envelope of the audio signals was estimated by using a filter bank that models the passage of the signal through the cochlea.^38^^−^ 40 Coherence between the speech envelope and neural activity in each voxel of both ROIs was obtained in the 0.5−10 Hz frequency band with 0.5 Hz frequency resolution.^7^, ^9^ The average coherence value of all voxels in LAC and RAC was calculated for each participant, condition, and frequency bin.

We performed group‐level statistics on the mean coherence values to identify frequency bands that showed significant speech–brain entrainment. For each condition, ROI (LAC and RAC), and frequency bin, speech coherence values were compared with surrogate coherence values using a two‐tailed permutation test (1000 permutations).^41^ Surrogate coherence values were obtained by computing coherence between the brain activity from the speech signals and the reversed speech signals.^3^ This provides an estimate of the coherence values expected by chance. For all these tests, we used a cluster‐based procedure to correct for multiple comparisons.^42^ Frequency bins within the significant cluster defined the frequency bands of interest.

Furthermore, we identified the maximum coherence (Coh_max_) value and the frequency (*f*_c_) value of the Coh_max_ for the frequency bands of interest, for each participant, condition, and ROI. For each frequency band of interest, separate ANOVAs were computed on the Coh_max_ and *f*_c_ values, with condition (decelerated, normal, and accelerated speech) and ROI (LAC versus RAC) as within‐subject factors. Before running the ANOVAs, we checked data normality using the Shapiro−Wilk test of normality.

## Cross‐frequency PAC analysis

We evaluated the coupling between the phase of the speech and the amplitude of neural oscillations in the AC.^3^ The speech envelope was bandpass filtered in 0.5 Hz steps between 0.5 and 10 Hz (fourth‐order Butterworth filter, forward and reverse, center frequency ±0.5 Hz). For each voxel in the ROIs (LAC and RAC), the activity was bandpass filtered in 0.5 Hz steps between 10 and 50 Hz (fourth‐order Butterworth filter, forward and reverse, center frequency ±0.5 Hz). The Hilbert transform was applied to the bandpass‐filtered signals to compute the phase dynamics of the speech and the amplitude dynamics of the activity in voxels within the ROIs. We computed MI between all combinations of phase (0.5–10 Hz) and amplitude (10–50 Hz) dynamics. MI was quantified using the direct method with quadratic extrapolation for bias correction, as described in the Information Breakdown Toolbox (see Ref. 43). Phase and amplitude signal dynamics were quantized into 10 equipopulated bins to build marginal and joint probability distributions. The average MI value of all the voxels in the LAC and RAC was calculated for each participant, condition, and phase/amplitude combination.

We performed group‐level statistics on the MI values to identify which frequency band combination showed significant PAC during speech processing. For each condition, ROI, and phase/amplitude combination frequency, speech MI values were compared with surrogate MI values using a two‐tailed permutation test (1000 permutations).^41^ Surrogate MI values were obtained by computing MI between brain activity from the speech and reversed speech signals.^3^ This provides an estimate of the MI values expected by chance. For all these tests, we used a cluster‐based procedure to correct for multiple comparisons.^42^ All frequency bins were included in the test.

Furthermore, we identified the maximum MI (MI_max_) value and the phase (*f*_phase_) and amplitude (*f*_amp_) frequency values of the MI_max_ for the MI clusters, for each participant, condition, and ROI. For each cluster, separate ANOVAs were computed on the MI_max_, *f*_phase_, and *f*_amp_ values, with condition (decelerated, normal, and accelerated speech), frequency band (delta and theta), and ROI (LAC versus RAC) as within‐subject factors. We checked data normality using the Shapiro−Wilk test of normality before running the ANOVAs.

## Results

**Figure 1:**
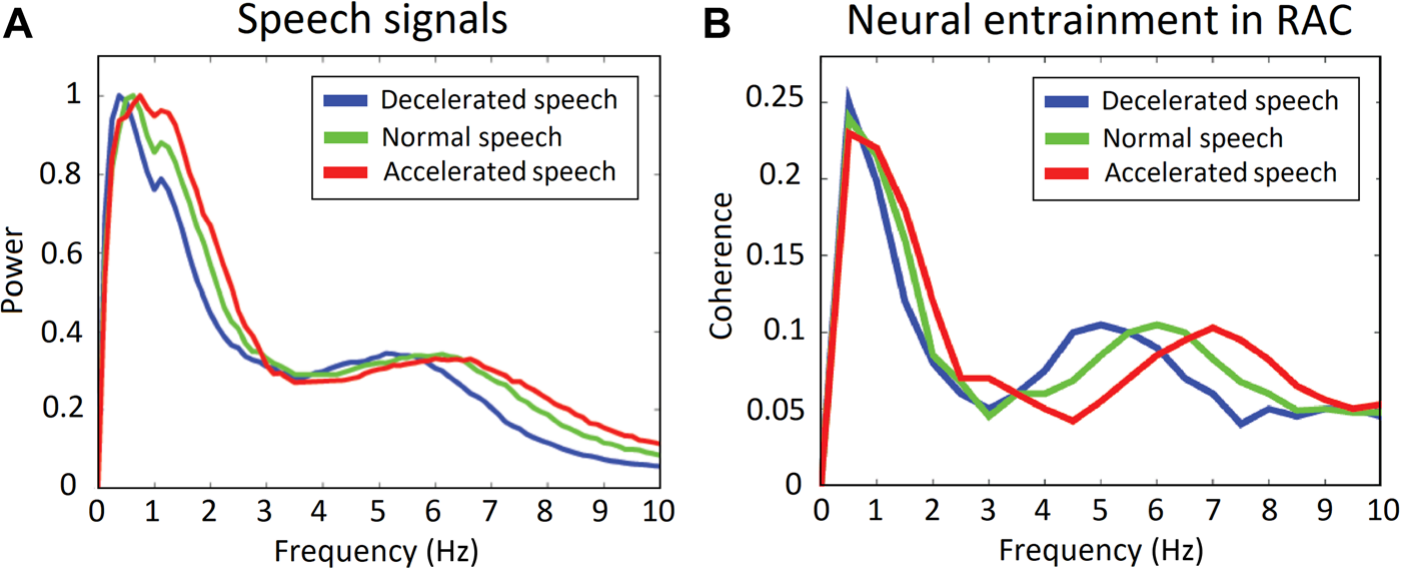
Neural entrainment analysis. (A) Power spectra of the decelerated (blue), normal (green), and accelerated (red) speech signals. (B) Coherence spectra for each condition in the right auditory cortex (RAC). We observed significantly higher coherence values for speech compared with surrogate data (*P* < 0.05, two‐sided permutation test, cluster corrected) in the 0.5−2 Hz and the 4−6.5 Hz bands for the decelerated speech, in the 0.5−2 Hz and the 4.5−7.5 Hz bands for the normal speech, and in the 0.5−2.5 Hz and 5.5−8.5 Hz for the accelerated speech.

The power spectrum of the normal speech envelope showed two main peaks within the delta (<3 Hz) and theta (4−7 Hz) frequency bands (Fig. 1A). The power spectrum of the normal speech envelope shifted to a lower frequency band for decelerated speech, and to a higher frequency band for accelerated speech (Fig. 1A).

### Neural entrainment analysis

In all conditions, we observed significantly higher coherence values for the speech compared with the surrogate data in the LAC and the RAC (Fig. 1B). Normal speech signals elicited significantly higher coherence values than did surrogate data in the 0.5−2 Hz (delta) band and the 4.5−7.5 Hz (theta) band (*P* < 0.05, two‐sided permutation test, cluster corrected). For decelerated speech, significantly higher coherence values (compared with surrogate data) were found in the 0.5−2 Hz (delta) band and the 4−6.5 Hz (theta) band (*P* < 0.05, two‐sided permutation test, cluster corrected). For accelerated speech, effects emerged in the 0.5−2.5 Hz (delta) band and in the 5.5−8.5 Hz (theta) band (*P* < 0.05, two‐sided permutation test, cluster corrected).

We identified the frequency of the maximum coherence (Coh_max_) value and the frequency (*f*_c_) value of Coh_max_ for the frequency bands of interest (delta and theta), for each condition and ROI (Table 1).

**Table 1:** Neural entrainment analysis

	Decelerated speech		Normal speech		Accelerated speech	
	LAC	RAC	LAC	RAC	LAC	RAC
Delta						
Coh_max_	0.18(0.09)	0.24(0.08)	0.18(0.07)	0.24(0.07)	0.17(0.08)	0.23(0.07)
*f*_c_	0.56(0.17)	0.53(0.12)	0.56(0.17)	0.59(0.2)	0.65(0.23)	0.71(0.36)
Theta						
Coh_max_	0.08(0.06)	0.11(0.06)	0.08(0.05)	0.1(0.04)	0.08(0.05)	0.11(0.04)
*f*_c_	4.79(0.25)	4.68(0.29)	5.62(0.26)	5.59(0.28)	6.65(0.27)	6.62(0.31)

note: Mean and standard error of the maximum coherence (Coh_max_) and frequency (*f*_c_) values for each condition (decelerated, normal, and accelerated speech), region (left auditory cortex (LAC) and right auditory cortex (RAC)), and frequency bands of interest (delta and theta bands).

In the delta band, the ANOVA on the Coh_max_ values showed a main effect of ROI (*F*(1,16) = 41.9, *P* < 0.01, $\eta_{p}^{2}$ = 0.72). A post hoc test (Bonferroni) showed that Coh_max_ values in the delta band were significantly higher in RAC than in LAC (*t* = 6.47, *P*_Bonferroni_ < 0.01, Cohen's *d* = 1.57). Results of the ANOVA for the *f*_c_ values in the delta band showed no main effect of condition (*F*(2,32) = 2.52, *P* = 0.1, $\eta_{p}^{2}$ = 0.14) nor ROI (*F*(1,16) = 2.13, *P* = 0.16, $\eta_{p}^{2}$ = 0.12).

**Figure 2:**
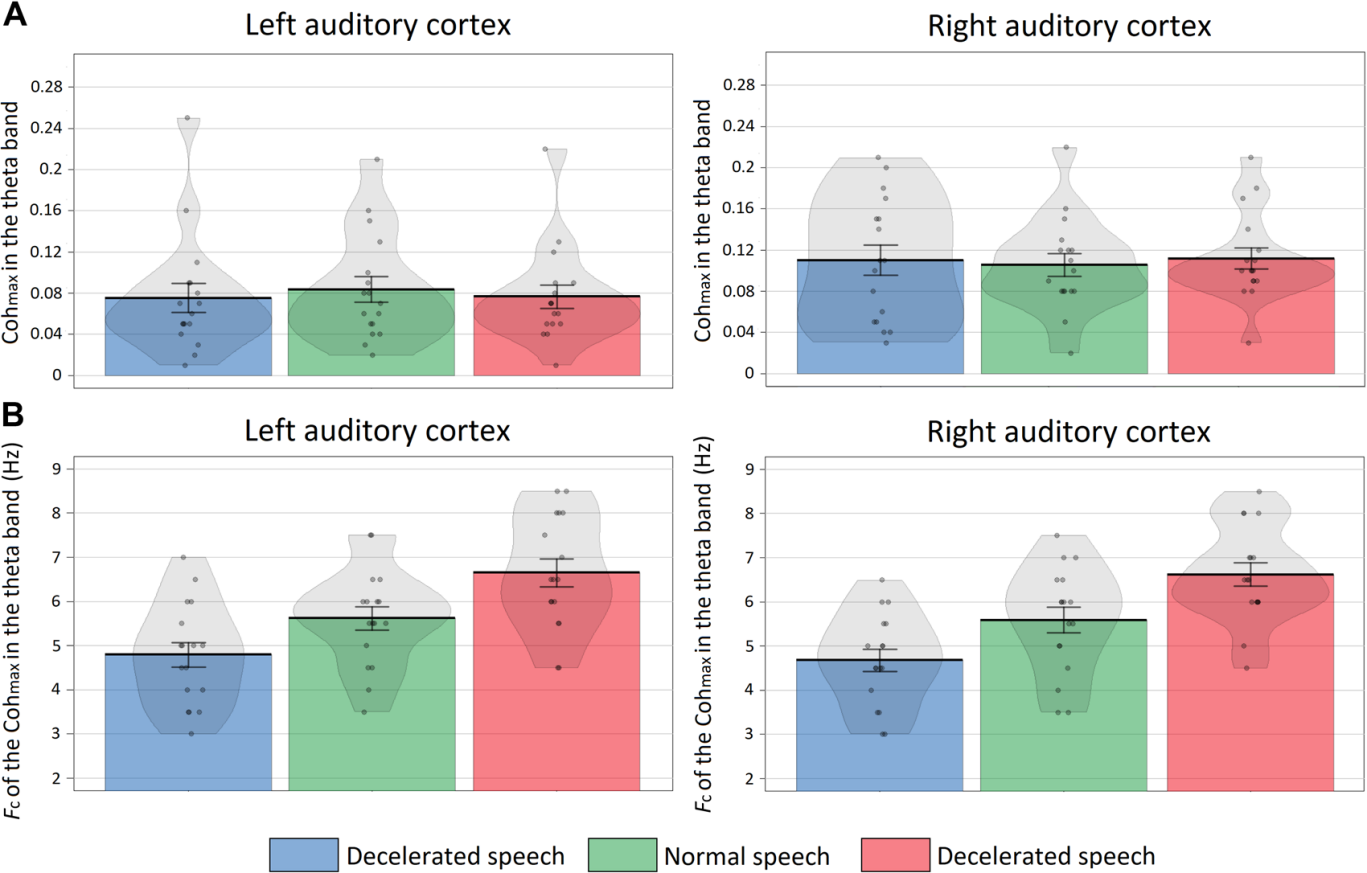
Neural entrainment in the theta band. (A) Maximum coherence value (Coh_max_) in the theta band for each condition (decelerated (blue), normal (green), and accelerate (red)) in the LAC and RAC. (B) The frequency of the Coh_max_ obtained for each condition in the LAC and RAC. Each dot represents the data of one participant and the shaded area shows the data distribution.

In the theta band, the ANOVA of the Coh_max_ values showed a main effect of ROI (*F*(1,16) = 6.43, *P* = 0.02, $\eta_{p}^{2}$ = 0.27) (Fig. 2A). A post hoc test showed that Coh_max_ values in the theta band were significantly higher in RAC than in LAC (*t* = 2.54, *P*_Bonferroni_ = 0.02, *d* = 0.59). Results of the ANOVA for *f*_c_ values in the theta band showed a main effect of condition (*F*(2,32) = 14.96, *P* < 0.01, $\eta_{p}^{2}$ = 0.48) (Fig. 2B). Post hoc tests showed that *f*_c_ values in the theta band were significantly higher in the normal compared with the decelerated speech condition (*t* = 3.25, *P*_Bonferroni_ = 0.02, *d* = 0.79), in the accelerated compared with the normal condition (*t* = 2.74, *P*_Bonferroni_ = 0.04, *d* = 0.066), and in the accelerated compared with the decelerated condition (*t* = 4.92, *P*_Bonferroni_ < 0.01, *d* = 1.19).

### Cross‐frequency PAC analysis

**Figure 3:**
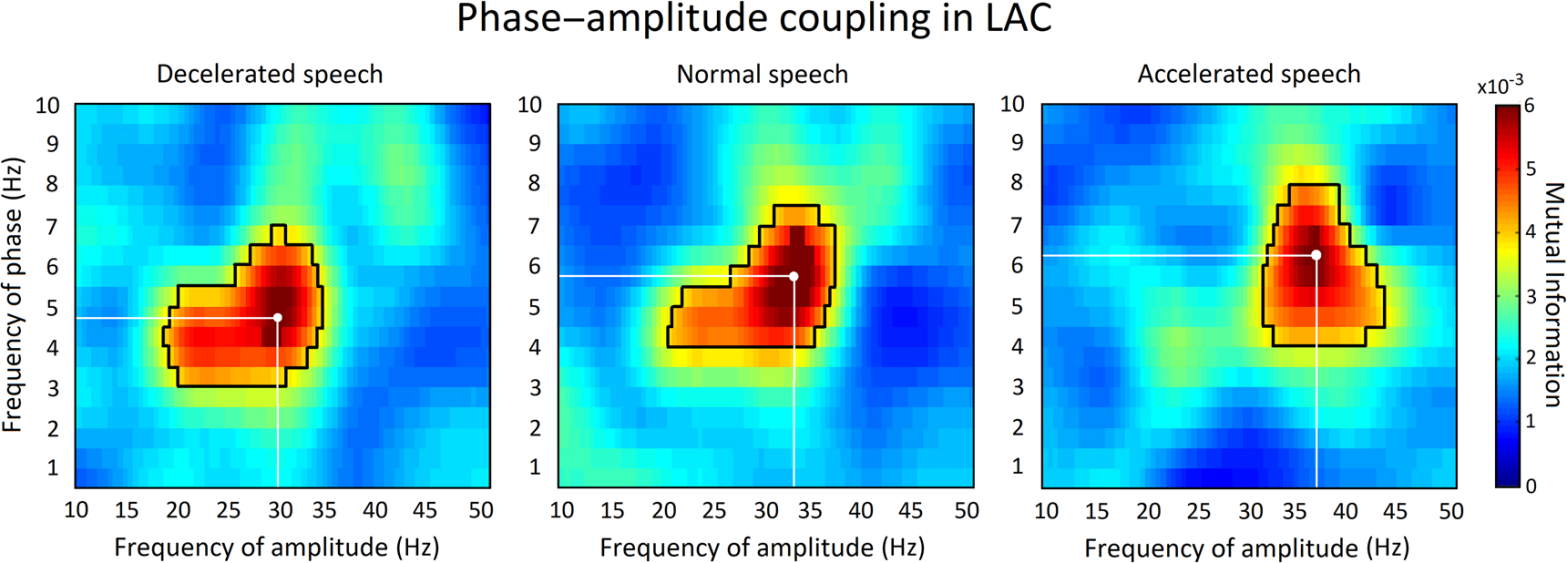
Cross‐frequency phase−amplitude coupling analysis. The spectral distribution of PAC for the decelerated (left), normal (middle), and accelerated (right) conditions in the LAC. Cross‐frequency PAC was quantified using mutual information (MI). For each condition, the black contour line encircles clusters showing significantly higher MI values for speech compared with surrogate data (*P* < 0.05, two‐sided permutation test, cluster corrected). The white dot represents the maximum MI value (MI_max_), and the white lines represent the phase (*f*_phase_) and amplitude (*f*_amp_) frequency values for MI_max_ within MI clusters.

In all conditions, MI values were significantly higher for the speech compared with the surrogate data in both LAC and RAC (Fig. 3). For the normal speech condition, we observed that the phase of neural oscillations in the ∼4−7.5 Hz (theta) band modulated the amplitude of neural oscillations in the ∼20−37 Hz (beta/gamma) band in LAC and RAC (*P* < 0.05, two‐sided permutation test, cluster corrected). For the decelerated speech condition, the phase of neural oscillations in the ∼3−6.5 Hz (theta) band modulated the amplitude of neural oscillations in the ∼18−34 Hz band in LAC and RAC (*P* < 0.05, two‐sided permutation test, cluster corrected). For the accelerated speech condition, the phase of neural oscillations in the ∼4−8 Hz (theta) band modulated the amplitude of neural oscillations in the ∼33−43 Hz (beta/gamma) band in LAC and RAC (*P* < 0.05, two‐sided permutation test, cluster corrected).

Furthermore, we measured the maximum MI value (MI_max_) and the phase (*f*_phase_) and amplitude (*f*_amp_) frequency values of MI_max_ for the MI cluster (theta‐beta/gamma), for each condition and ROI (Table 2).

**Table 2:** Cross‐frequency phase−amplitude coupling analysis

	Decelerated speech		Normal speech		Accelerated speech	
	LAC	RAC	LAC	RAC	LAC	RAC
Theta‐beta/gamma						
MI_max_ (×10^−3^)	5.96(0.53)	6.06(0.54)	6.1(0.44)	6.07(0.54)	6.06(0.59)	5.94(0.54)
*f*_phase_ (Hz)	4.74(0.26)	4.65(0.25)	5.53(0.24)	5.68(0.25)	6.41(0.33)	6.44(0.31)
*f*_amp_ (Hz)	29.12(1.31)	28.94(1.22)	32.76(1.43)	32.65(1.62)	37.88(1.61)	38.12(1.82)

note: Mean and standard error of the maximum mutual information (MI_max_) and the phase (*f*_phase_) and amplitude (*f*_amp_) frequency values for each condition (decelerated, normal, and accelerated speech), region (left auditory cortex (LAC) and right auditory cortex (RAC)), and MI cluster (theta‐beta/gamma).

**Figure 4:**
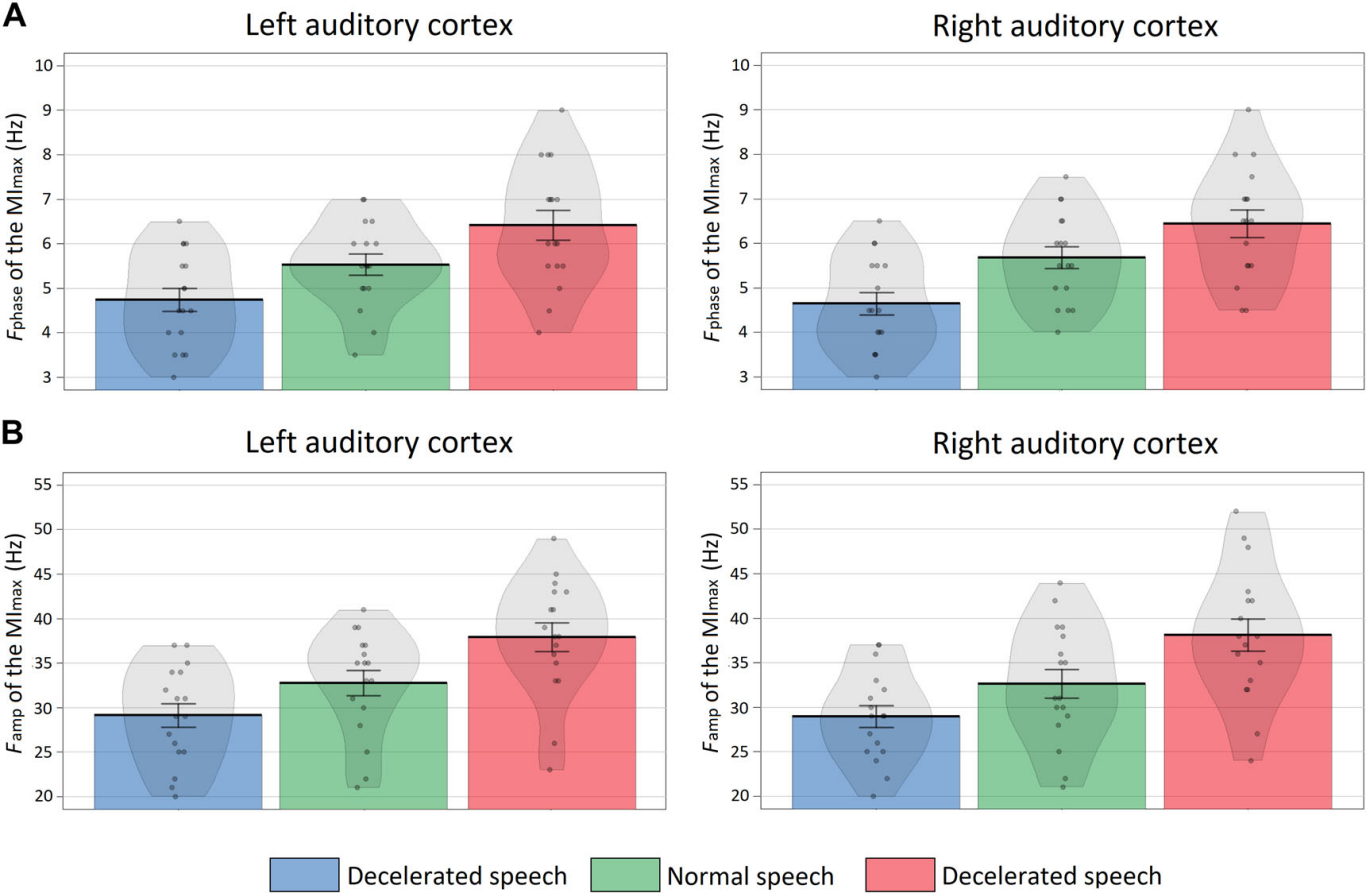
Theta/gamma phase−amplitude coupling. Phase (*f*_phase_) and amplitude (*f*_amp_) frequency of the maximum mutual information (MI_max_) value for the theta/gamma clusters obtained for each condition (decelerated (blue), normal (green), and accelerated (red)) in the LAC and RAC. Mean and standard error of the *f*_phase_ and *f*_amp_ values are plotted. Each dot represents the data of one participant and the shaded area shows the data distribution.

The ANOVA of the MI_max_ values showed no main effect of condition (*F*(2,32) = 0.01, *P* = 0.99, $\eta_{p}^{2}$ < 0.01) nor ROI (*F*(1,16) < 0.01, *P* = 0.93, $\eta_{p}^{2}$ < 0.01). Results of the ANOVA for the *f*_phase_ values showed a main effect of condition (*F*(2,32) = 3.04, *P* < 0.01, $\eta_{p}^{2}$ = 0.71) (Fig. 4A). Post hoc tests showed that *f*_phase_ values were significantly higher in the normal than the decelerated speech condition (*t* = 2.5, *P*_Bonferroni_ = 0.05, *d* = 0.62), in the accelerated than the normal speech condition (*t* = 3.14, *P*_Bonferroni_ = 0.02, *d* = 0.76), and in the accelerated than the decelerated speech condition (*t* = 4.44, *P*_Bonferroni_ < 0.01, *d* = 1.08). Results of the ANOVA for the *f*_amp_ values showed a main effect of condition (*F*(2,32) = 15.77, *P <* 0.01, $\eta_{p}^{2}$ = 0.49) (Fig. 4B). Post hoc tests showed that *f*_amp_ values were significantly higher in the normal than the decelerated speech condition (*t* = 2.8, *P*_Bonferroni_ = 0.05, *d* = 0.62), in the accelerated than the normal speech condition (*t* = 3.84, *P*_Bonferroni_ < 0.01, *d* = 0.93), and in the accelerated than the decelerated speech condition (*t* = 5.27, *P*_Bonferroni_ < 0.01, *d* = 1.28).

## Discussion

While listening to the natural speech, oscillatory neural activity in low‐frequency bands synchronizes with the syllabic and phrasal patterns of the input (respectively, theta: 6−8 Hz, and delta: <2 Hz). This synchronization accurately adapts to the speech rate, as reported in the present study and in the previous studies.^24^^−^ 27 Indeed, we observed that the frequency of the maximum coherence peaks in the theta frequency band aligns to the speech rate in bilateral auditory cortices. Importantly, such low‐frequency synchronization impacts the way lexically ambiguous words, including sounds with an intermediate duration between a minimal phonetic pair, are interpreted.^26^ Why does low‐frequency entrainment impact the “phonemic disambiguation” effect observed by these authors? Our data suggest that theta band oscillatory activity has a direct influence on high‐frequency phonemic processing, through hierarchical cross‐frequency PAC. Here, we evaluated the sensitivity of this hierarchical cross‐frequency PAC to the temporal envelope pattern of the auditory speech input. This neural phenomenon has been interpreted as a key mechanism by which high‐frequency neural oscillatory activity (gamma: ∼30−40 Hz) related to acoustic sampling can be modulated by slower oscillatory mechanisms sensitive to the coarse‐grain syllabic structure of the stimulus.^3^, ^14^ This cross‐frequency interaction is stronger for forward than for backward speech, revealing its tight relationship to intelligible speech processing.^3^

While auditory cortical entrainment in the theta band has been shown to adapt to the timing of the input in a number of studies,^24^^−^ 27 frequency‐domain adaptation by the nested gamma component to the speech rate had yet to be investigated. Theta−gamma coupling varies its strength across time depending on the content of the speech input. Gross *et al*.^3^ showed that theta−gamma PAC is enhanced at speech edges (periods of increased acoustic energy after segments of silence) during natural speech listening. This finding suggests that the timing of this high‐frequency phenomenon is tightly related to the presence/absence of relevant information in the speech input. However, this finding did not constitute conclusive evidence for speech rate−dependent modulation in the frequency domain of theta−gamma coupling. It could be argued that gamma activity does not adapt to the rate of the speech stimulus, but instead retains a fixed response around 35 Hz at any speech rate (based on proposals that consider the auditory gamma response to be a high‐level response not directly related to sensory input^29^, ^30^). This interpretation would suggest that theta−gamma coupling is not directly involved in perceptual decoding of acoustic input but could, among other possibilities, reflect activation of abstract phonemic representations. Alternatively, gamma activity might exhibit varying peaks in the frequency domain, depending on the rate of the speech input. This would support the idea that this high‐frequency component is involved in tracking the acoustics of the speech input.^14^ Our data support this second alternative.

We analyzed the whole set of nested oscillatory patterns in response to different speech rates and found that the amplitude envelope of gamma shares maximum MI with the phase of theta at various frequencies, which depend on the speech rate. As discussed in Gross *et al*.,^3^ the observed theta−gamma PACs suggest a model for how the brain takes advantage of the coarse‐grain temporal structure of the speech envelope to select “windows of interest.” In these time windows, the neurocognitive system can deploy gamma band resources for optimally “processing” fine‐grain temporal information in speech, such as phonemes. If this neural correlate reflects a proper acoustic sampling of such fast speech components, it should also adjust as speech input accelerates or decelerates. The frequency‐peak modulation of the nested gamma band that we report here supports this latter view. In particular, gamma band activity “released” in the relevant (theta range) time intervals resonates with the fine‐grain acoustic properties of the input, thus internally tracking phonetic speech properties. This internal representation of an external stimulus would provide the basis for higher level activation of related abstract phonemic information.^14^ This kind of higher level process could be identified in beta band oscillatory activity related to the comprehension and not related to the acoustic properties of speech input.^27^ In addition, our data support the idea that only gamma‐related processing of this fine‐grain information is temporally coupled in the auditory regions during processing of syllabic speech rhythms. The human auditory system has to prioritize specific (low frequency) rhythms to support a finer analysis of acoustic signals (faster rhythms). Indeed, the demultiplexing idea could explain how our auditory perceptual system copes with many rhythms simultaneously in the same time period (for a more detailed proposal on microcircuitry, see Ref. 14).

The present findings are in line with the asymmetric sampling in time (AST) proposal advanced by Giraud and Poeppel,^2^ which identified coupling between low‐ and high‐frequency oscillations as a fundamental processing mechanism for optimal speech sampling. An important aspect of the AST model concerns the division of labor between the two hemispheres: while the RAC is considered to be mainly involved in tracking slow speech rhythms, the homologous left region should be mainly involved in sampling phonemic information. These different computations would, respectively, support paralinguistic processes (such as speaker identification) tracked as low rhythms by the right hemisphere and linguistic processes (such as phoneme and syllable recognition) tracked by the left hemisphere. Although this model accounts well for the set of findings, we report here, we did not observe any hemispheric specialization in gamma activity. The nested gamma activity highlighted in this study was not reliably different for the two auditory cortices.^44^ Consequently, it is not possible to conclude that the two auditory cortices have such a clear‐cut frequency‐dependent division of labor.^45^ While the low‐frequency cortical tracking shows its maximum peak in the right hemisphere (as predicted by the AST model), the opposite is not necessarily true for the high‐frequency speech processing component, which is equally visible in both auditory cortices.

We did not observe relevant effects in the delta band response. Speech−brain coupling in delta did not show any statistically robust modulation dependent on the experimental manipulation. This could be due to methodological factors since the frequency resolution we could explore was limited to 0.5 Hz and this cannot exclude the possibility that the delta entrainment peak showed lower frequency variations across the three conditions. More important for our claims, we did not find clear evidence of PAC involving the delta band. This could indicate that the delta band speech tracking component is not as sensitive to the acoustic properties of speech as other frequency bands (theta and gamma), and possibly suggest it is more related to endogenous language−related processes.^9^

The frequency‐adaptive nested gamma response to the fine‐grain structure of the auditory signal could explain experimental evidence suggesting that auditory cortical entrainment influences word recognition.^26^, ^28^ If the nested high‐frequency gamma response adapts to the fine‐grain structure of the speech signal, it is possible that ongoing faster (or slower) low‐frequency entrainment to faster (or slower) speech input directly affects the perception of words that have ambiguous meanings due to the intermediate duration of a key phoneme. If this phoneme is sampled at a higher frequency (faster speech rates), it may be perceived as having a longer duration, whereas if it is sampled at a lower frequency (slower speech rates), it may be perceived as having a shorter duration. This would better explain how low‐frequency cortical entrainment affects word disambiguation, by pointing directly to how coarse‐grain speech−brain coupling can modulate fine‐grained phonemic sampling.

Related to this point, it may be the case that syllabic rates in speech play a significant role in the development of phonemic identification and categorization.^46^ Interestingly, participants suffering from phonological developmental dyslexia have been shown to exhibit sluggish auditory attentional shifting skills at speeds falling within the theta rate.^47^^−^ 50 This could reflect a deficit in disengaging attentional focus from one syllabic unit sufficiently rapidly to process the next unit in the speech stream. Such impairments have been suggested to generate noise in speech inputs entering the focus of attention (and the perceptual integration window), hindering the extraction of precise phonemic information.^51^ The present results suggest that slowing down speech when speaking to individuals with atypical phonological development may allow more time for their sluggish attentional systems to engage and disengage from syllabic rhythms, and, in turn, facilitate the extraction of precise and stable fine‐grain phonemic information.

## Conclusion

With the present data, we bring new evidence concerning the nature of gamma auditory activity during natural speech listening and reveal its fine‐grained adaptive nature, by focusing on oscillatory patterns of neuronal activity reconstructed in the AC. Relying on previous evidence that low‐frequency oscillations in the auditory regions adapt to the rate of external speech,^26^ we made a step forward by demonstrating that this effect relies on nested gamma activity whose frequency peak changes depending on speech rate. Overall, the fact that high‐frequency oscillations are modulated by the external speech rate supports the role of this gamma mechanism in sampling the fine‐grain temporal information present in speech. Theta−gamma coupling seems to be a crucial component of the initial encoding process necessary for speech comprehension. Recent proposals also stress the importance of characterizing the internal mechanisms involved in internally reconstructing external input in order to better understand higher level abstract processes.^22^ Oscillatory activity in the delta and beta band could be, respectively, involved in syntactic structuring of the input and the deployment of rapid online predictions regarding incoming information.^10^, ^27^ The functional relation of these latter endogenous components to theta−gamma coupling has yet to be determined but could explain the limited comprehension capacity seen at compression rates higher than the ones explored in the present study.

## Competing interests

The authors declare no competing interests.

## Supporting information


**Figure S1**. Selection of the regions of interest (ROIs). Brodmann areas 41 (red) and 42 (blue) were selected as ROIs. The brain slice in the axial plane (*Z* = 12, 14, 15, and 16 in the MNI coordinates) illustrates the deepness of the ROIs. BA41 and BA42 in the left hemisphere of the MNI brain are also included.
