# Supplementary figures and images for: Phase−amplitude coupling between theta and gamma oscillations adapts to speech rate
Source: Ann N Y Acad Sci. 2019 Apr 24;1453(1):140–52. doi: 10.1111/nyas.14099 (PMC6850406; doi:10.1111/nyas.14099)

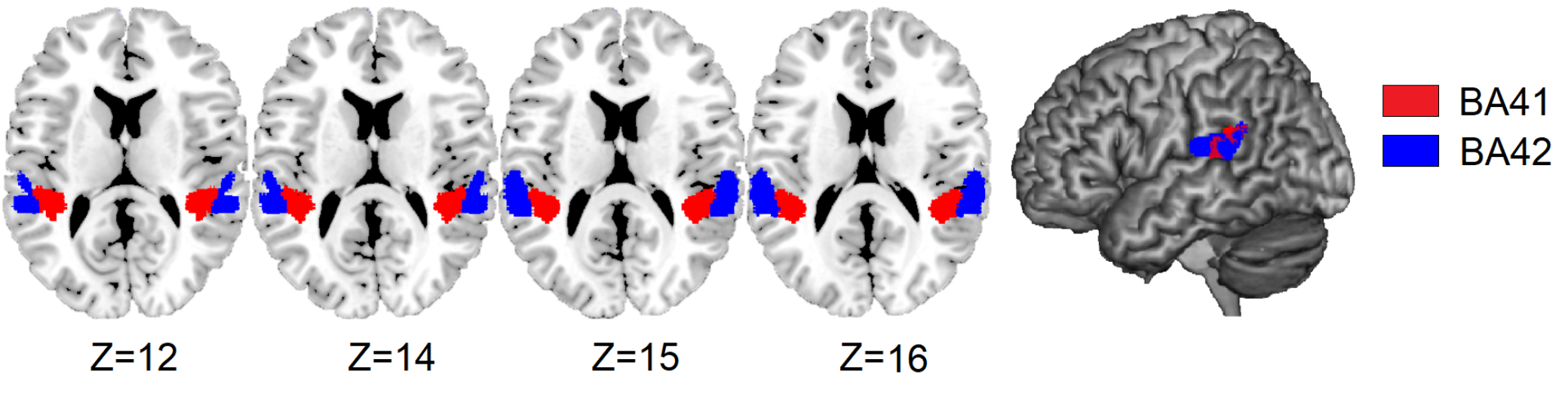

Supplement: Supplementary file 1 — Figure S1. Selection of the regions of interest (ROIs). Brodmann areas 41 (red) and 42 (blue) were selected as ROIs. The brain slice in the axial plane (Z = 12, 14, 15, and 16 in the MNI coordinates) illustrates the deepness of the ROIs. BA41 and BA42 in the left hemisphere of the MNI brain are also included. [file NYAS-1453-140-s001.tif]
